# Myocardial Deformation in Fontan Patients Assessed by Cardiac Magnetic Resonance Feature Tracking: Correlation with Function, Clinical Course, and Biomarkers

**DOI:** 10.1007/s00246-021-02650-w

**Published:** 2021-07-27

**Authors:** Alessia Callegari, Simona Marcora, Barbara Burkhardt, Michael Voutat, Christian Johannes Kellenberger, Julia Geiger, Emanuela Regina Valsangiacomo Buechel

**Affiliations:** 1 Division of Pediatric Cardiology, Pediatric Heart Center, University Children’s Hospital Zurich, Steinwiesstrasse 75, 8032 Zürich, Switzerland; 2 Paediatric Cardiology Unit, Papa Giovanni XXIII Hospital, Bergamo, Italy; 3 Division of Cardiac Surgery, Pediatric Heart Center, University Children’s Hospital Zurich, Zürich, Switzerland; 4 Department of Diagnostic Imaging, University Children’s Hospital Zurich, Zürich, Switzerland; 5 Children’s Research Center, Zürich, Switzerland; 6 University of Zurich, Zürich, Switzerland

**Keywords:** Fontan, CMR-FT, CMR, Strain, Biomarkers

## Abstract

Cardiac MR (CMR) is a standard modality for assessing ventricular function of single ventricles. CMR feature-tracking (CMR-FT) is a novel application enabling strain measurement on cine MR images and is used in patients with congenital heart diseases. We sought to assess the feasibility of CMR-FT in Fontan patients and analyze the correlation between CMR-FT strain values and conventional CMR volumetric parameters, clinical findings, and biomarkers. Global circumferential (GCS) and longitudinal (GLS) strain were retrospectively measured by CMR-FT on Steady-State Free Precession cine images. Data regarding post-operative course at Fontan operation, and medication, exercise capacity, invasive hemodynamics, and blood biomarkers at a time interval ± 6 months from CMR were collected. Forty-seven patients underwent CMR 11 ± 6 years after the Fontan operation; age at CMR was 15 ± 7 years. End-diastolic volume (EDV) of the SV was 93 ± 37 ml/m^2^, end-systolic volume (ESV) was 46 ± 23 ml/m^2^, and ejection fraction (EF) was 51 ± 11%. Twenty (42%) patients had a single right ventricle (SRV). In single left ventricle (SLV), GCS was higher (*p* < 0.001), but GLS was lower (*p* = 0.04) than in SRV. GCS correlated positively with EDV (*p* = 0.005), ESV (*p* < 0.001), and EF (*p* ≤ 0.0001). GLS correlated positively with EF (*p* = 0.002), but not with ventricular volumes. Impaired GCS correlated with decreased ventricular function (*p* = 0.03) and atrioventricular valve regurgitation (*p* = 0.04) at echocardiography, direct atriopulmonary connection (*p* = 0.02), post-operative complications (*p* = 0.05), and presence of a rudimentary ventricle (*p* = 0.01). A reduced GCS was associated with increased NT-pro-BNP (*p* = 0.05). Myocardial deformation can be measured by CMR-FT in Fontan patients. SLVs have higher GCS, but lower GLS than SRVs. GCS correlates with ventricular volumes and EF, whereas GLS correlates with EF only. Myocardial deformation shows a relationship with several clinical parameters and NT-pro-BNP.

## Introduction

Treatment of patients with a single ventricle (SV) consists of a staged procedure, the last step being the Fontan operation [1]. Thanks to the significant developments in the surgical, interventional and medical techniques, survival has dramatically improved in the last decades [1]. Better survival demasks the morbidity occurring during long-term follow-up in these patients, whose circulation is demanded to function based on unphysiological hemodynamics [1, 2]. During the staged surgical procedures, the SV myocardium is exposed to different pressure- and volume loading conditions. These may lead to unfavorable ventricular remodeling and to diastolic and/or systolic dysfunction, and eventually to cardiac failure and clinical deterioration [1, 3]. Therefore, effective monitoring of myocardial function and timely introduction of medical therapy may help improving prognosis.

In Fontan patients echocardiography presents some important limitations due to the abnormal geometry of the SV and to a frequently limited acoustic window [4, 5]. Cardiac magnetic resonance (CMR) is recommended during imaging follow-up in Fontan patients [6, 7]. Strain measurements as parameters of myocardial deformation have been reported in Fontan patients by using echocardiography [2, 4, 8]. More recently, CMR feature-tracking (CMR-FT) is increasingly used for measuring strain in Fontan patients; however, more data are necessary to demonstrate the clinical significance of the measurements [3, 4, 9–11].

The aim of this study was to assess the feasibility of CMR-FT in Fontan patients and to evaluate potential correlation between strain values measured by CMR-FT and clinical data such as patient characteristics, post-operative complications, hemodynamic measures, and blood biomarkers.

## Methods

### Patients

All Fontan patients who underwent a CMR examination in our institution between January 2014 and October 2018 were retrospectively reviewed. Exclusion criteria were insufficient image quality (*n* = 3) and missing informed consent (*n* = 3). Electronic records of the patients were reviewed regarding medical history and clinical data at follow-up, i.e., in a time interval of ± 6 months from the CMR examination. These included post-surgical complications, medication, blood biomarkers, exercise capacity and hemodynamic parameters invasively acquired during cardiac catheterization, if available. Echocardiographic reports were reviewed for ventricular function and presence of atrioventricular valve regurgitation (AVVR). Global ventricular contraction was qualitatively described in three categories: normal = 1, moderately decreased = 2, clearly impaired = 3. The degree of AVVR was graded I (absent or minimal) to IV (severe).

### Cardiac Magnetic Resonance and Feature Tracking

All CMR examinations were performed with a 1.5T scanner (Signa HDxt and MRI 450, GE Medical Systems, Milwaukee, WI, USA) using a 32-channel phased array cardiac coil. For measuring ventricular function, steady-state free precession (SSFP) cine images were acquired in a horizontal and vertical long-axis plane, as well in a short-axis plane covering the entire single ventricle. The SSFP parameters were as follows: retrospective cardiac gating, 40 phases/cardiac cycle, TE 1.5–1.8 ms, TR 2.8–3.1 ms, flip angle 45°, bandwidth 125 kHz, matrix 224 × 224, number of excitations 1, field of view 250–350, views per segment 4–10 according to heart rate. In-plane resolution was 1–1.5 mm and, true temporal resolution was < 25 ms.

Myocardial strain was measured by applying feature tracking on SSFP cine images during post-processing with a dedicated software (Qstrain, Medis Version 3.3, Leiden, Netherlands). Circumferential strain (CS) was measured on short-axis images and longitudinal strain (LS) was measured on long-axis images. The endocardial borders of the SV were first segmented manually in the end-diastolic phase and subsequently expanded to all phases using an automatic border detection algorithm. The endocardial borders were checked for adequacy in all cardiac phases and manually corrected if necessary. The software provided endocardial peak systolic strain.

Measurement of radial strain has previously not been recommended due to inaccuracy and variability of the measurements [12]. Measurement of global circumferential strain (GCS) has been recommended at mid-ventricular level [13]. In our cohort, we performed preliminary analysis of 40 patients and have observed a large variability of the measurements at the basal or apical level, but more robust data at mid-ventricular level. Thus, all values of GCS used for the study were taken at mid-ventricular level. Reference values for both LV [14] and RV [15] are available for adults, while pediatric reference values are available only for the LV [16].

### Statistics

Continuous variables are expressed as mean ± SD, categorical variables in counts and percentages. Groups were compared using unpaired *t* tests; Levene’s test for equality of variance was used to analyze if the variance in the two groups was significantly different. Differences in categorical data were evaluated with contingency tables and *χ*^2^ tests. The correlation among continuous variables was tested using Pearson’s correlations. Intraobserver variability was tested by repeating CMR-FT analysis in all patients by the same observer at a time interval of 3 months. Inter-observer variability was assessed by repeating strain measurements in 10 patients by two observers blinded to each other. Repeatability of CMR-FT measurements was then expressed with intraclass correlation coefficient. Statistical significance was defined by values of *p* < 0.05. As values of NT-pro-BNP were markedly skewed, a logarithmic transformation was performed and used in all statistical tests involving this parameter.

Statistical analysis was performed with the software SPSS 25.0.0 (Spss Inc, IBM Company, Chicago Illinois, USA).

The study protocol was approved by the local ethics authorities (KEK-ZH-Nr.2017-00566) and adheres to the declaration of Helsinki (version 2013).

## Results

### Patient Characteristics

A total of 47 patients (28 males) fulfilled the inclusion criteria and were enrolled in the study. CMR was performed at a mean age of 15 ± 7 years, weight 50 ± 22 kg, and height 150 ± 22 cm. The time interval between the Fontan operation and CMR was 11.6 ± 6.2 years. Diagnoses consisted of Hypoplastic Left Heart Syndrome (HLHS) in 15 (32%), Double Inlet Left Ventricle in 13 (27%), Pulmonary Atresia in 10 (21%), Double Outlet Right Ventricle in 4 (8%), Tricuspid Atresia in 3 (6%), and Double Inlet Right Ventricle and Ebstein Anomaly in one patient each (2%). The first surgical palliation consisted of a shunt in 30 (65%) and pulmonary banding in 14 patients (30%). A Norwood procedure was performed in 14 patients (30%). Six patients (13%) with HLHS were first palliated with a hybrid approach, consisting of a bilateral pulmonary banding and ductus stenting). As next step 31 (66%) patients underwent a cavopulmonary connection, 7 (15%) received a hemi-Fontan procedure, 6 (13%) a comprehensive stage I–II. Type of surgery at stage II was unknown in 3 patients (6%).

The Fontan operation was eventually performed at the age of 37 ± 17 months, a weight of 14 ± 3 kg, and a transcutaneous oxygen saturation (SpO_2_) of 82 ± 5%. A total cavopulmonary connection was created in 44 (94%) patients, consisting of 41 (87%) extracardiac conduits and 3 (6%) lateral tunnels. A fenestration was made in 22 of the 44 cases (50%). The remaining 3 (6%) patients received a direct atriopulmonary connection. Hospitalization length was 23 ± 10 days. At least one complication occurred in 19 (40%) patients; including chylothorax in 6 (13%), diaphragmatic paralysis in 6 (13%), thromboembolic event in 1 (2%), arrhythmia (defined as need for antiarrhythmic drug therapy at discharge, pacemaker or ablation) in 2 (4%), significant bleeding in 1 (2%), and prolonged effusions in 2 (4%).

### CMR Results

The results of ventricular volumes and function as well as global strain values measured by CMR-FT are shown in Table 1. Single right ventricles (SRV) were larger than single left ventricles (SLV) (*p* = 0.02) and presented a lower global contractility (*p* = 0.05). In 28 (60%) cases, a rudimentary second ventricle was visible. In 5 (11%) cases a Fontan fenestration was still present at time of CMR. Short-axis images could be acquired in all 47 patients, while long-axis images were available in 37 patients. Insufficient image quality was observed in 3/47 short-axis images and 4/37 the long-axis images. Thus, GCS measurements were performed in 44 patients, GLS in 33 patients.

**Table 1:** Ventricular volumes and strain measurements obtained by CMR-FT for all ventricles and according to dominant anatomy

	All	LV (*n* = 25)	RV (*n* = 20)	*p
EDV (ml/m^2^)	93 ± 37	78 ± 28	99 ± 51	0.06
ESV (ml/m^2^)	46 ± 23	37 ± 14	52 ± 31	**0.02**
Stroke volume (ml/m^2^)	47 ± 19	41 ± 13	49 ± 26	0.1
EF (%)	51 ± 11	55 ± 10	48 ± 13	**0.05**
GLS (%)	− 16.3 ± 4.3%	− 14.7 ± 3.2	− 18.0 ± 4.9	**0.04**
GCS (%)	22.2 ± 3.4%	− 23.6 ± 2.6	− 20.0 ± 3.4	** < 0.001**

Bold values are statistically significant

Data presented as mean ± SD

*EDV* end-diastolic volume, *ESV* end-systolic volume, *EF* ejection fraction, *GLS* global longitudinal strain, *GCS* global circumferential strain, *LV* left ventricle, *RV* right ventricle

*p refers to the comparisons between LV and RV

**Fig. 1:**
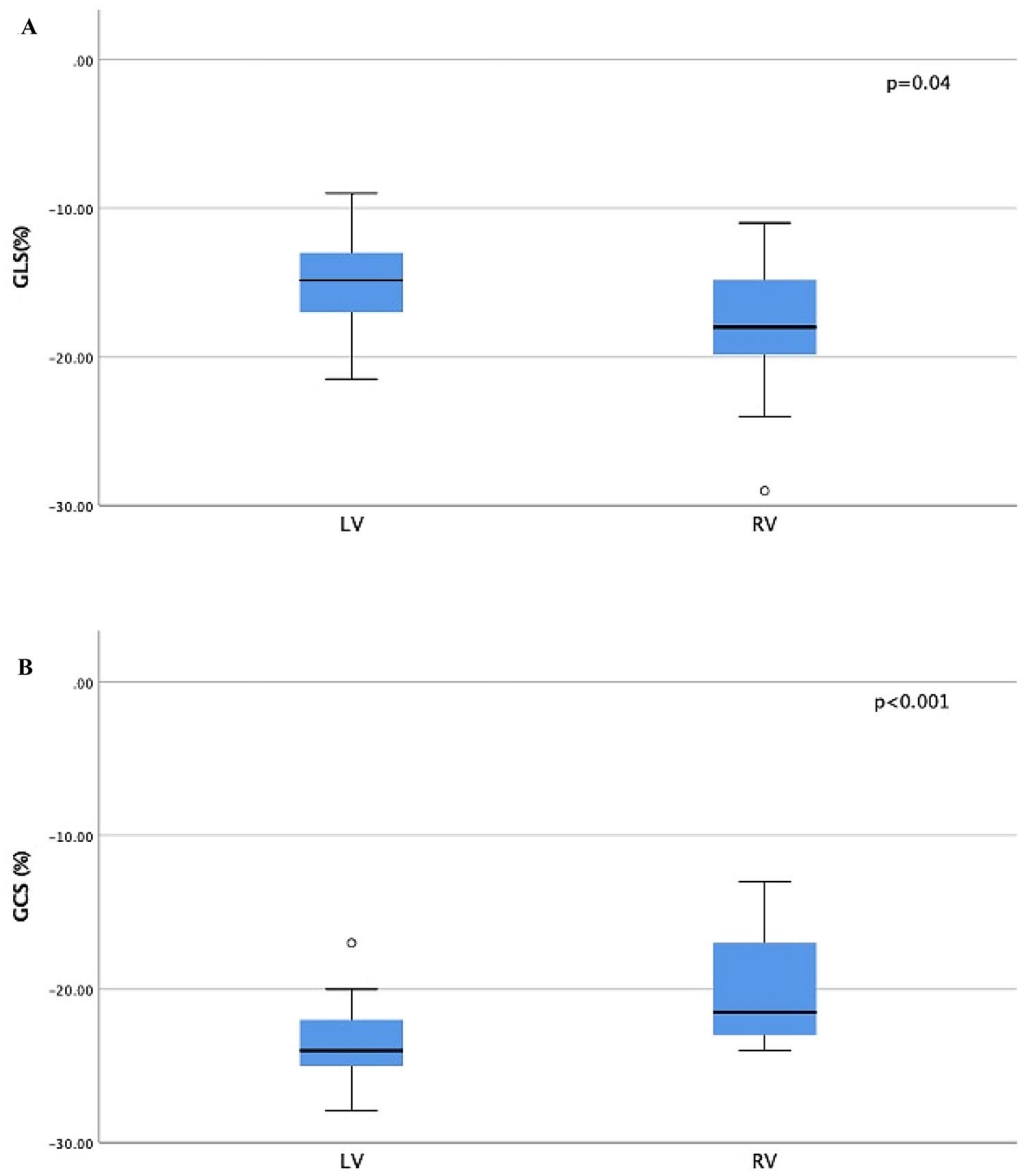
Box plot showing the difference in strain according to ventricular anatomy. **A** Global longitudinal strain (GLS%) in left ventricle (LV) and right ventricle (RV)*.* **B** Global circumferential strain (GCS%) in left ventricle (LV) and right ventricle (RV)

Mean total GCS was − 22.2 ± 3.4%. Total global longitudinal strain (GLS) was − 16.3 ± 4.3%. GLS was better (= lower value) in SRV than in SLV, while GCS was better in SLV (Fig. 1).

The intraclass correlation coefficient for intra-observer variability was 0.93 (0.84–0.96) for GCS and 0.95 (0.87–0.97) for GLS. Inter-observer reproducibility presented an intraclass correlation coefficient for GCS of 0.88 (0.80–0.97), for GLS of 0.97 (0.88–0.99).

### Clinical Data

Clinical follow-up data were available at a time interval of ± 4 ± 4.8 months from CMR in 43 patients. Twenty-two patients (45%) were in functional class NYHA I, 16 (34%) in NYHA class II, 3 (6%) in NYHA class III, and one in NYHA class IV. Five patients (11%) presented with a diaphragmatic paralysis, 2 (4%) with a scoliosis, one (2%) with protein losing enteropathy, and one (2%) with plastic bronchitis. Cardiac medication was taken by 14 patients (30%) and included diuretics in 11 (13%), ACE inhibitors in 5 (11%), and beta-blockers in 2 (4%). Echocardiography showed a normal ventricular function (grade 1) in 26 (55%), a moderately impaired function (grade 2) in 14 (30%), and a severely impaired function (grade 3) in 4 (9%) cases. Sinus rhythm was present in 37 (79%), a junctional or atrial rhythm in 9 (19%) patients.

Hemodynamic cardiac catheterization data were available in 10 patients (time interval from CMR ± 4 ± 4.8 months). In the overall group mean pulmonary artery pressure was 15 ± 3 mmHg, pulmonary capillary wedge pressure 12 ± 2 mmHg, and end-diastolic ventricular pressure 11 ± 2 mmHg.

Cardiopulmonary exercise test data were available in 14 patients (30%); mean peak oxygen consumption (peakVO_2_) was 25 ± 10 ml/kg/min. Heart rate was elevated at rest (100 ± 16 bpm) and increased well during exercise (170 ± 21 bpm). Mean SpO_2_ at rest was 92 ± 1.8% and declined at peak exercise by an average of 5%.

Blood biomarkers were available in 26 patients (55%) (time interval from CMR 2.5 ± 2.5 months). Mean total protein was 69 ± 10 g/l, albumin 45 ± 7 g/dl, creatinine 56 ± 18 μmol/l, NT-pro-BNP 231 (1160) ng/l, and alanine aminotransferase 33 ± 11 IU/l. All parameters were within normal ranges.

### Correlations Between Myocardial Deformation and Volumetric and Clinical Data

**Fig. 2:**
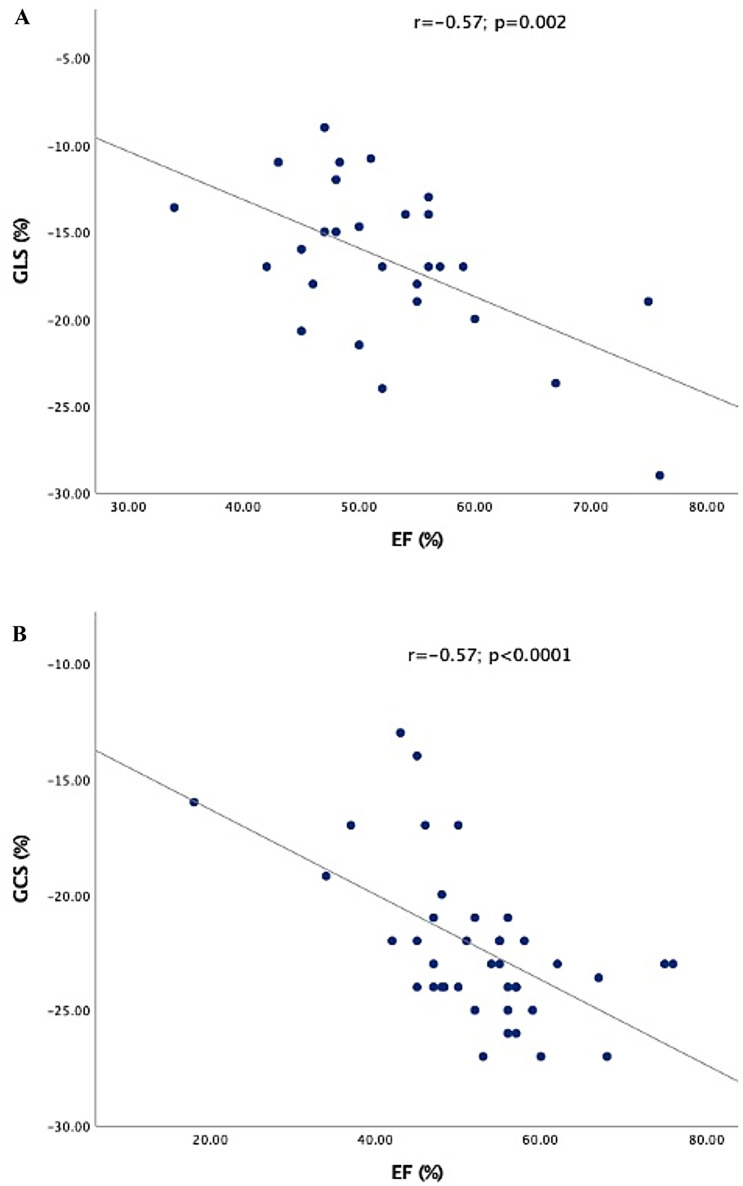
Scatter plot and linear regression correlation between strain and ejection fraction. **A** Global longitudinal strain (GLS%) and Ejection fraction (EF%). **B** Global circumferential strain (GLS%) and Ejection fraction (EF%)

**Fig. 3:**
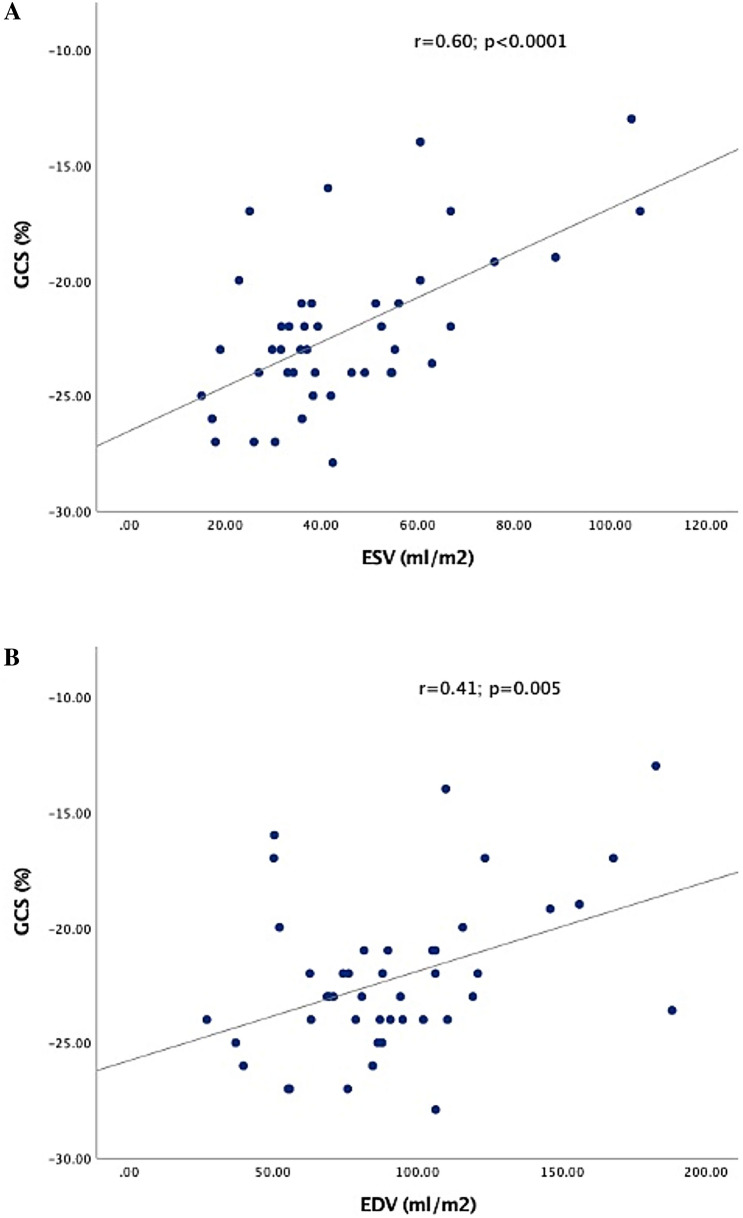
Scatter plot and linear regression correlation between global circumferential strain (GCS%) and ventricular volumes. **A** Global longitudinal strain (GLS%) and End-systolic Volume (ESV ml/m^2^). **B** Global circumferential strain (GLS%) and End-diastolic Volume (EDV ml/m^2^)

Myocardial deformation correlated well with ventricular volumes and contractility independently from ventricular morphology (Table 2). Positive correlation was observed between strain and EF (Fig. 2). GCS decreased (= higher values) with enlarging ventricular volumes (Fig. 3). Higher NT-pro-BNP values correlated weekly with decreased (= higher values) GCS (Table 2). In contrast GLS was independent from ventricular volumes and NT-pro-BNP (Table 2).

**Table 2:** Correlations between CMR volumetric, NT-pro-BNP, and strain measurements for all ventricles

	GCS (%)		GLS (%)	
	*r*	*p*	*r*	*p*
CMR volumetric				
EF (%)	− 0.57	< 0.0001	− 0.57	**0.002**
EDV (ml/m^2^)	0.41	0.005	− 0.06	0.7
ESV (ml/m^2^)	0.60	< 0.0001	0.15	0.41
Stroke volume	0.09	0.5	0.29	0.1
Clinical data				
NT-pro-BNP (ng/L)	0.38	0.05	0.2	0.3

Bold value indicates statistically significant

*EDV* end-diastolic volume, *ESV* end-systolic volume, *EF* ejection fraction, *GCS* global circumferential strain, *GLS* global longitudinal strain, *AVVR* atrioventricular regurgitation

GCS values were worse (= higher) in patients with a rudimentary ventricle (*p* = 0.01), an atriopulmonary connection (*p* = 0.02), post-surgical complications (*p* = 0.05), previous chylothorax (*p* = 0.03), echocardiographically reduced ventricular function (*p* = 0.003), and significant AVVR (*p* = 0.04). In contrast, GLS did not correlate with any clinical parameters.

Strains were not related to any other parameter, including demographics, time interval from Fontan operation, perioperative data at Fontan surgery, number of cardiac interventions, arrhythmias, invasive hemodynamic values, other blood values, functional class, exercise capacity, and need for medication.

## Discussion

CMR-FT is a novel post-processing tool enabling measurement of myocardial deformation on cine CMR images. CMR-FT is not limited by restricted ultrasound acoustic window after repeated cardiac surgery and can be applied independently from the underlying ventricular morphology and/or a complex ventricular geometry. The role of CMR-FT as a sensitive imaging modality for detecting subclinical myocardial dysfunction and for potentially predict clinical outcome, has been reported for some acquired and congenital cardiac diseases [12, 17–20]. This study demonstrates that CMR-FT is feasible and reproducible in pediatric Fontan patients and adds important novelty in describing the correlation between CMR-FT measurements and various clinical parameters. CMR-FT is easy to measure on conventional SSFP cine images and its post-processing requires a limited amount of time.

Our results are in line with other previously published data (Table 3) reported for Fontan patients and particularly those published by Ghelani et al. in a larger but similar patient population [3, 4, 9, 11, 18].

**Table 3:** Summary of previously published studies on CMR-FT in single ventricle patients

Paper	Ventricle		Age (year)	CMR-FT^£^		RV versus LV		Correlation		No correlation	Reproducibility
	RV	LV		GCS	GLS	GCS	GLS	GCS	GLS		
Schmidt 2014 [4]	\	13	26	\	\	\	\	PeakVO_2_ NYHA Age at Fontan		Interval from Fontan CMR: EF, ESV, EDV	Good
Ghelani 2016 [9]	60	74	16	− 18.8 ± 5.5	− 14.6 ± 4.4	\	\	\	\	2D speckle tracking	Good
Kato 2017 [3]	8	13	14	LV − 18.9 ± 2.6 RV − 14.9 ± 4 controls − 24.6 ± 3	LV − 13.9 ± 3.5 RV − 13.4 ± 3.0 controls − 17 ± 4.8	RV < LV RV < controls	LV < controls LV = RV	Markers of fibrosis		Echo	\
Salehi 2018 [10]	55	\	5*	\	− 19.8 (− 27.4; − 13.3)	RV < in septum	\	\	CMR: EF	Demographics NYHA Heart rate, SpO_2_	Good
Ghelani 2018 [18]	70	74	16	LV − 25 (− 29; − 20) RV − 21 (− 14; − 17)	LV − 17 (− 19; − 13) RV − 18 (− 21; − 14)	RV < LV	RV = LV	AVVR Survival EDV	AVVR	Demographics PeakVO_2_ Cardiac catheter data	\
Hu 2018 [11]	\	19	9	LV − 14.55 ± 3.79	LV − 18 ± 4.61	\	\	Reduced to controls EF	\	GLS = controls	Good
Our study	20	23	15	LV − 24.6 ± 2.6 RV − 20.0 ± 3.4	LV − 14.7 ± 3.2 RV − 18.0 ± 6.8	RV < LV RV < in septum RV < septal time to peak	RV > LV RV > in apex	CMR: EF, ESV, EDV Function at echo AVVR Complications TCPC Rudimental ventricle NT-pro-BNP	CMR: EF	Fenestration NYHA Arrhythmia Age at Fontan Hospitalization length Demographics Interval from Fontan PeakVO_2_ Cardiac catheter data	Good

Results in terms of mean ± SD/medians (IQR)

\ not available*, LV* left ventricle, *RV* right ventricle, *CMR-FT* cardiac magnetic resonance feature tracking, *GCS* global circumferential strain, *GLS* global longitudinal strain, *EDV* end-diastolic volume, *ESV* end-systolic volume, *EF* ejection fraction, *AVVR* atrioventricular regurgitation, *TCPC* total cavopulmonary connection

*Patients at stage II and III of palliation

GCS and GLS values of the SLV in Fontan patients are impaired (higher values) in comparison to the reference CMR-FT strain values for the LV in healthy pediatric subjects, reported as − 24 ± 2.4% for GCS and − 15.5 ± 1.9% for GLS [16]. In contrast, strain values of the SRV are better than those reported by our group and others for the systemic RV [19, 20]. The presence of a to a certain degree developed LV beside the single RV may have a major influence om myocardial mechanics. So, in HLHS the presence of a developed LV is a risk factor for worse prognosis [21, 22].

The different myofiber architecture between the SLV and SRV is a valuable explanation for the differences observed in the myocardial deformation of the SVs with better (lower values) GLS in SRV patients and better GCS in SLV patients. The RV presents with a predominant layer of longitudinally oriented fibers that lead to a strong longitudinal contraction [18, 23, 24]. Similarly to other investigations on the systemic RV [19], our findings suggest that the SRV may not able to totally remodel like a LV, due to the lack of a robust circumferential fiber layer in the wall structure of the RV.

The correlation between strain and ventricular volumes and ejection fraction is quite intuitive and indicates the strong intrinsic relationship between myocardial mechanics and ejection of blood [10, 11].

Regarding ventricular volumes, we have observed that GCS decreases (higher values) when the SV enlarges; in contrast values of GLS are not changing with ventricular size. Ghelani et al. reported a similar observation in their Fontan cohort [18]. Ventricular contraction may be primarily determined by concentric (circumferential) myofibers contraction, and the myocardium of a stretched ventricle may first present with decreased circumferential deformation rather than with decreased longitudinal deformation. This may result in a stronger correlation between ventricular volumes and GCS rather than with GLS.

Even though other studies have reported the use and feasibility of CMR-FT in Fontan patients (Table 3), we are the first group analyzing the correlation between CMR-FT strain values and clinical data, biomarkers, and echocardiographic findings during follow-up. Our results provide novel and interesting data. Elevated NT-pro-BNP values are correlated with decreased GCS. Thus NT-pro-BNP may represent an additional prognostic parameters if combined with echocardiographic and CMR imaging parameters [25].

Additional factors associated with decreased GCS were palliation with an atriopulmonary connection and post-operative chylothorax. It is well known that one of the possible causes for chylothorax in Fontan patients is an abnormally increased systemic venous pressure; suboptimal hemodynamics with elevated filling pressures after the Fontan operation may have a negative influence on myocardial deformation.

## Limitations

The retrospective design of this study is its most important limitation. The study population may present with a selection bias, since not all Fontan patients at our institution received a routine CMR examination in the past. The small number of available clinical follow-up data impeded a robust statistical analysis for risk factors. Our results give some suggestions about the potential clinical implications of CMR-FT, but its real prognostic value needs to be assessed in larger and prospective follow-up studies. As normal values for CMR-FT measurements in SVs are not available, CMR-FT may be more useful for serial evaluation of each patient during follow-up, by using each patient as his own control. Ghelani et al. have reported composite imaging parameters, echocardiographic and CMR data, as strong predictor for outcome in Fontan patients [25]. Our results may suggest adding strain parameters obtained by CMR-FT and biomarkers such as NT-Pro-BNP.

## Conclusions

CMR-FT enables reproducible measurements of global myocardial strain in Fontan patients. Strain values correlate with ventricular volume, EF, NT-Pro-BNP as biomarkers for cardiac failure, post-operative chylothorax, and reduced contractility and AVVR at echocardiography. SRVs have better GLS but worse GCS than SLVs.

These preliminary data suggest a potential relationship between imaging parameters and clinical markers for prognosis and may help designing future prospective CMR follow-up studies. As CMR-FT analysis does not prolong the duration of examination, we advocate to add FT to every CMR performed in Fontan patients.
